# 

**Published:** 1941

**Authors:** 


### The Bristol Medico-Chirurgical Journal

A JOURNAL OF THE MEDICAL SCIENCES FOR THE
WEST OF ENGLAND AND SOUTH WALES.

PUBLISHED UNDER THE AUSPICES OF
*THE BRISTOL MEDICO-CHIRURGICAL SOCIETY.*

EDITOR:
J. A. NIXON, C.M.G., M.D., F.R.C.P.

WITH WHOM ARE ASSOCIATED
E. W. HEY GROVES, D.Sc., M.S., F.R.C.S.
A. E. ILES, O.B.E., M.B., B.S., F.R.C.S.
A. RENDLE SHORT, M.D., B.S., B.Sc., F.R.C.S.
W. MELVILLE CAPPER, M.B., B.S., F.R.C.S.
T. M. LING, M.D., M.R.C.P.
E. WATSON-WILLIAMS, M.C., Ch.M., F.R.C.S., Assistant Editor

"Scire est nescire, nisi id me

Scire alius sciret."

*VOL. LVIII.*

BRISTOL: J. W. ARROWSMITH LTD.

### Contents of Vol. LVIII.

*Page*

Thyrotoxicosis. By A. Rendle Short, M.D., B.S., B.Sc., F.R.C.S. 1

The Importance of the First Year of War in Mental Disease. By R. E. Hemphill, M.D., D.P.M. .. .. .. .. .. .. 11

Sex Hormones and Blood Pressure. By A. Guirdham, D.M., B.Sc., D.P.M. .. .. .. .. .. .. .. .. .. 19

Medicine from Ancient Greece to Soviet Russia. By Professor B. Farrington, M.A. .. .. .. .. .. .. .. 45

Experiences of First Aid in Air Raids .. .. .. .. 57

The Casualty Surgery of Air Raids .. .. .. .. .. .. 74

Reviews of Books .. .. .. .. .. .. .. .. 22, 91

Editorial Notes .. .. .. .. .. .. .. .. 27, 95

Obituaries .. .. .. .. .. .. .. .. 31, 97

The Medical Library of the University of Bristol .. 32, 98

Meetings of Societies .. .. .. .. .. .. .. .. 101

Publications Received .. .. .. .. .. .. 35, 101

Local Medical Notes .. .. .. .. - .. 36, 102

### The Medical Library of the University of Bristol

WITH WHICH IS INCORPORATED

**The Library of the Bristol Medico-Chirurgical Society**

*The following donations have been received since the publication of the list in November,* 1940.

May, 1941.

| | |
|---|---|
| Bristol Royal Infirmary (1) | 9 volumes. |
| General Medical Council (2) | 2 volumes. |
| Professor E. W. Hey Groves (3) | 10 volumes. |
| Michell Clarke Fund (4) | 32 volumes. |
| Middlesex Hospital Medical School (5) | 1 volume. |
| Otago University (6) | 1 volume. |
| Smithsonian Institution (7) | 1 volume. |
| Mr. E. Watson-Williams (8) | 12 volumes. |
| Messrs. John Wright & Sons Ltd. (9) | 1 volume. |

Unbound periodicals have also been received from Dr. R. F. Barbour, Dr. H. C. Bristowe, Professor E. W. Hey Groves, Professor C. Bruce Perry, Dr. G. de M. Rudolf, Dr. J. Odery Symes, and Mr. E. Watson-Williams.

### THE ONE HUNDRED AND SEVENTY-THIRD LIST OF BOOKS

The figures in round brackets refer to the figures after the names of the donors. The books to which no such figures have been attached have either been bought from the Library Fund or received through the *Journal*.

**Albee, F. H.** .. .. *Bone-graft Surgery in Disease, Injury and Deformity* .. (3) 1940

*Analecta Therapeutica* .. (8) 1939

**Armstrong, H. G.** .. *Principles and Practice of Aviation Medicine* (4) 1939

**Bailey, H. H.** .. .. *Emergency Surgery* .. 4th Ed. (9) 1940

**Bernard, L., and others** *Les abcès du poumon après tonsillectomie* .. (8) 1932

**Blacklock, T. B., and Southwell, T.** *Guide to Human Parasitology* 4th Ed. (4) 1940

**Blond, K.** .. *Hæmorrhoids and their Treatment* .. 1940

**Boyd, W.** .. *Pathology of Internal Diseases* .. 3rd Ed. (4) 1940

**Brailsford, J. F.** .. .. *The Radiology of Bones and Joints* 2nd Ed. (4) 1935

*British Pharmacopœia*, 1932, *Third Addendum* .. 1941

**Brünings, W.** .. .. *Die direkte Laryngoskopie, Bronchoskopie und Oesophagoskopie* .. (8) 1910

**Cade, S.** .. *Malignant Disease and its Treatment by Radium* (3) 1940

**Cameron, A. T.** .. *Textbook of Biochemistry* .. 5th Ed. (4) 1938

**Canuyt, G. et Rozier, J.** *L'anesthésie locale et régionale en Oto-Rhino-Laryngologie* .. (8) 1920

*Catalogue of Kodak Medical Film Library* .. (8) 1933

**Conybeare, J. J.** (Ed.) *Textbook of Medicine* .. 5th Ed. 1941

**Covington, E.** .. .. *The Efficient Dental Assistant* .. (4) 1940

**Cuvier, G.** .. *The Animal Kingdom* .. New Ed. (8) 1851

**Davidson, L. S. P., and Anderson, I. A.,** *A Textbook of Dietetics* .. 1940

**Duke-Elder, W. S.** .. *Textbook of Ophthalmology* .. Vol. III, (4) 1940

**Ewing, J.** .. *Neoplastic Diseases* .. 4th Ed. (4) 1940

**Fairbrother, R. W.** .. *Textbook of Bacteriology* .. 3rd Ed. (4) 1940

**Frazer, J. E.** .. *Manual of Embryology* .. 2nd Ed. (4) 1940

**Gaddum, J. H.** .. .. *Pharmacology* .. 1940

**Grossman, L. I.** .. .. *Root Canal Therapy* .. (4) 1940

**Hurst, Sir A.** .. .. *Medical Diseases of War* .. (4) 1940

**Hutchison, R., and Moncrieff, A.** *Lectures on Diseases of Children* Ed. 8 (4) 1940

**Hutton, I. E.** .. .. *Hygiene of Marriage* .. 8th Ed. (4) 1940

**Illingworth, C. F. W., and Dick, B. M.** *A Textbook of Surgical Pathology* 4th Ed. 1941

**Kaplan, I. I.** .. .. *Radiation Therapy* .. (4) 1937

**Katz, L., and others** .. *Handbuch der speziellen Chirurgie des Ohres* 2nd Ed. (8) 1913

**Klotz, A.** .. *Les accidents mortels de l'anesthésie locale* .. (8) 1929

**Lewis, T.** .. *Soldier's Heart and the Effort Syndrome* 2nd Ed. (4) 1940

**MacCullum, W. G.** .. *Textbook of Pathology* .. 7th Ed. (4) 1940

**Macintosh, R. R., and Pratt, F. B.** *Essentials of General Anæsthesia* .. (4) 1940

**Macleod, J. M. H., and Muende, I.** *Pathology of the Skin* .. 2nd Ed. (4) 1940

**Maxson, L. H.** .. .. *Spinal Anæsthesia* .. (4) 1938

**Milligan, W.** .. .. *The Rise and Progress of Laryngology* .. (8) 1922

**Ogilvie, W. H.** (Ed.) .. *Lancet Primer on Wound Infection* .. (4) 1940

**Osborne, J.** .. .. *Dental Mechanics for Students* .. (4) 1940

**Pack, G. T., and Livingston, E. M.** (Ed.). *Treatment of Cancer and Allied Diseases* .. .. .. .. .. .. 3 Vols. (3) 1940
**Parfitt, J. R., and Herbert, W. E.** *Operative Dental Surgery* 4th Ed. (4) 1939
**Perkins, G.** .. .. .. *Fractures* .. .. .. .. .. .. .. .. .. .. .. .. (3) 1940
**Pickworth, F. A.** .. *The Pathology of the Nasal Sinuses* .. .. (8) 1932
**Pohle, E. A.** (Ed.) .. *Clinical Roentgen Therapy* .. .. .. .. .. (4) 1938
**Prinz, H., and Greenbaum, S. S.** *Diseases of the Mouth and their Treatment* 2nd Ed. (4) 1939
**Rawling, L. B.** .. .. *Landmarks and Surface Markings of the Human Body* .. .. .. .. .. .. .. .. 8th Ed. 1940
**Robson, J. M.** .. .. *Recent Advances in Sex and Reproductive Physiology* .. .. .. .. .. .. .. .. 2nd Ed. (4) 1940
**Rolleston, Sir H.** (Ed.) *British Encyclopædia of Medical Practice, Index* (4) 1941
**Rose and Carless** .. *Manual of Surgery* .. 16th Ed., 2 Vols. (4) 1940
**Roussy, G.** .. .. .. *Cancer* .. .. .. .. .. .. .. .. .. .. .. .. .. .. 1940
**Samuels, S. S.** .. .. *Diseases of Peripheral Arteries* .. .. 2nd Ed. 1940
**Scudder, J.** .. .. .. *Shock* .. .. .. .. .. .. .. .. .. .. .. .. .. .. (4) 1940
**Shanks, S. C., and others** (Ed.). *Textbook of X-ray Diagnosis* Vol. III 1939
**Souttar, H. S.** .. .. *The Art of Surgery* .. .. 4th Ed., 2 Vols. (3) 1940
**Tilley, H.** .. .. .. *Etude critique du traitement des sinusites fronto-ethmoidales* .. .. .. .. .. .. .. .. .. .. (8) 1921
*Treatment in General Practice* .. .. .. .. .. .. .. .. Vol. IV (3) 1940
**Watson-Jones, R.**.. .. *Fractures and other Bone and Joint Injuries* 2nd Ed. (3) 1941
**Watson-Williams, P.** .. *Diagnostic Exploral Suction Sampling of the Paranasal Sinuses*.. .. .. .. .. .. .. .. .. (8) 1934
**Whitby, L. E. H., and Britton, C. J. C.** *Disorders of the Blood* 3rd Ed. (4) 1939
**Whitwell, J. R.** .. .. *Syphilis in Earlier Days* .. .. .. .. .. .. 1940
**Wiener, A. S.** .. .. *Blood Groups and Blood Transfusion* 2nd Ed. (4) 1939
**Wright, S.** .. .. .. *Applied Physiology* .. .. .. .. .. 7th Ed. 1940

### TRANSACTIONS, REPORTS, JOURNALS, ETC.

Annual Report of the Smithsonian Institution, 1939 .. .. .. .. .. (7) 1940
Bristol Royal Infirmary Annual Reports, 1806–1870 ; 1884–1903 ; 1925 ; 1927 ; 1938 .. .. .. .. .. .. .. .. .. .. .. .. .. .. .. .. (1) 1807–1939
Collected Papers of the Middlesex Hospital Medical School, 1939–40 (5) 1941
Dentists' Register, 1941 .. .. .. .. .. .. .. .. .. .. .. .. .. .. (2) 1941
Medical Directory, 1941 .. .. .. .. .. .. .. .. .. .. .. .. .. .. .. 1941
Medical Register, 1941 .. .. .. .. .. .. .. .. .. .. .. .. .. .. .. (2) 1941
Proceedings of the University of Otago Medical School, No. 18, 1941 (6) 1941
Quarterly Cumulative Index Medicus, Vol. 27, Jan.–June, 1940 .. .. 1940
Transactions of the Ophthalmological Society of the United Kingdom, Vol. LX .. .. .. .. .. .. .. .. .. .. .. .. .. .. .. .. .. .. .. .. 1940
Yearbook of Radiology, 1940 .. .. .. .. .. .. .. .. .. .. .. .. .. 1940

### Publications Received

From General Medical Council :

*Third Addendum to the British Pharmacopoeia,* 1932.

From Messrs. Hamish Hamilton Medical Books Ltd. :

*The Nursing Couple.* By Merell P. Middlemore, M.D. Price 7s. 6d.

From Messrs. William Heinemann (Medical Books) Ltd. :

*A Complete Outline of Fractures* (*including Fractures of the Skull*). By J. Grant Bonnin, M.B., B.S. (Melbourne), F.R.C.S. (Eng.), M.R.C.O.G. Price 25s.

From Messrs. H. K. Lewis & Co. Ltd. :

*Treatment in General Practice—Surgery.* Vol. IV. Articles republished from the *British Medical Journal.* Price 16s.

From Messrs. E. & S. Livingstone :

J. F. Sutherland's *First Aid to Injured and Sick.* Revised and re-written by Halliday Sutherland, M.D. Price 6d.

From Oxford University Press :

*Pharmacology.* By J. H. Gaddum, Sc.D., M.R.C.S., L.R.C.P. Price 17s. 6d.

*Fractures.* By George Perkins, M.C., M.Ch.Oxon., F.R.C.S. Price 20s.

*A Practical Manual of Diseases of the Chest.* By Maurice Davidson, M.A., M.D. (Oxon.), F.R.C.P. (Lond.). Second Edition. Price 42s.

*Principles and Practice of Diphtheria Immunization.* By J. Tudor Lewis, M.D. (Lond.). D.P.H. Price 8s. 6d.

*Gynaecological Operations.* By J. Lyle Cameron, M.D., F.R.C.S. (Eng.), F.A.C.S., M.R.C.O.G. Price 21s.

From Messrs. John Wright & Sons Ltd. :

*British Journal of Surgery.* No. 111. January, 1941. Price 12s. 6d.

*An X-ray Atlas of Silicosis.* By Arthur J. Amor, M.D. (Lond.), M.Sc. (Wales). With translation of the legends into French by Robert E. Horne, M.A. Price 30s.

### Local Medical Notes

THE Thirtieth Long Fox Memorial Lecture was delivered at 8.30 p.m. on Tuesday, June 24th, 1941, in the H. H. Wills Physics Theatre (Royal Fort), Bristol University, by Dr. H. H. Carleton, M.D., F.R.C.P. The Vice-Chancellor, Dr. T. Loveday, presided, with Professor A. Rendle Short (Chairman Long Fox Appointing Board), Mr. E. Watson-Williams (Honorary Treasurer and Secretary), and seven other members of the Appointing Board and an audience of fifty-one persons. The lecturer chose as his subject: "The Methods of Science: Origins, Successes and Limitations."

**Conjoint Board.**—L.R.C.P., M.R.C.S.—*Final Examination. Pass:* J. M. Joshua (Pathology and Midwifery), J. H. G. Morris (Midwifery), J. I. Williams (Medicine).

L.D.S., F.R.S.—*Final Examination. Pass:* J. F. Williams.

### Bristol Medico-Chirurgical Society.

### President.

C. CORFIELD, M.D. Durh.

### President-Elect.

### Committee.

*Ex-Officio* { J. A. NIXON, *C.M.G.*, B.A., M.D. Cantab., F.R.C.P. (Editor of *Journal*).

(Vacant), (Hon. Librarian).

*Elected.*

L. A. MOORE, M.B., Ch.B. Brist. (Ex-President).
R. C. CLARKE, *O.B.E.*, M.B., Ch.B.Brist.
H. H. CARLETON, M.A., M.D., B.Ch.Oxon.
A. L. TAYLOR, M.D. Lond.
P. S. MARTIN, *M.C.*
T. MILLING, M.B., Ch.B. Belf.
W. V. WOOD, *M.C.*, B.A. Cantab.
H. L. SHEPHERD, M.B., Ch.M. Brist.
W. A. JACKMAN, F.R.C.S.

### Honorary Secretary.

R. V. COOKE, Ch.M.

### Honorary Treasurer.

A. E. ILES, *O.B.E.*, M.B., B.S. Lond., F.R.C.S.

### University Library Sub-Committee.

A. R. SHORT, B.Sc., M.D. Lond., F.R.C.S.
C. BRUCE PERRY, M.D. Brist., F.R.C.P.
H. J. DREW SMYTHE, *M.C.*, M.S., M.D. Lond., F.R.C.S., F.C.O.G.

Medical Practitioners wishing to join the Society are requested to communicate with the Hon. Secretary, Mr. R. V. Cooke, Failand Lodge, Guthrie Road, Clifton. The Annual Subscription is £1 1s., payable to Hon. Treasurer.

---

*Extracts from Journal By-laws.*

4.—The *Journal* shall be delivered free to Subscribers and also to Members of the Society whose subscriptions are not in arrear.

6.—The Annual Subscription to the *Journal* is 9/–, payable to Hon. Treasurer.

---

Communications intended for insertion in the *Journal* should be sent to the Editor, Dr. J. A. Nixon, 7 Lansdown Place, Clifton, Bristol.

Books for Review and Exchange Journals should be sent to the Assistant Editor, *Bristol Medico-Chirurgical Journal*, Medical Library, The University, Bristol. Reviews should be returned with the books to Dr. T. M. Ling, 50 Pembroke Road, Bristol 8.

Communications referring to the delivery of the *Journal* should be sent to the Hon. Treasurer, Mr. A. E. Iles, 87 Pembroke Road, Bristol 8.

Cases for Binding Vols. I—LVII are now ready, and may be obtained price 2/6 each (postage extra), upon application to J. W. Arrowsmith Ltd., Quay Street, Bristol; or the *Journal* will be bound, including Case, for 7/– per volume. The inclusive index for Volumes I—L may be obtained, price 3/– (postage extra).

### Bristol Medico-Chirurgical Society.

### PAST PRESIDENTS.

1874—75. FREDERICK BRITTAN, M.D., B.S. Dub.
1875—76. ROBERT WILLIAM COE.
1876—77. SAMUEL MARTYN, M.D. Ed.
1877—78. AUGUSTIN PRICHARD, M.D. Berlin.
1878—79. HENRY EDWARD FRIPP, M.D. Würzburg.
1879—80. WILLIAM MICHELL CLARKE.
1880—81. JOSEPH GRIFFITHS SWAYNE, M.D. Lond.
1881—82. EDWARD LONG FOX, M.D. Oxon.
1882—83. JAMES GEORGE DAVEY, M.D. St. And.
1883—84. WILLIAM JOHNSTONE FYFFE, M.D., B.A. Dub.
1884—85. GEORGE FORSTER BURDER, M.D. Aberd.
1885—86. WILLIAM HENRY SPENCER, M.D., M.A. Cantab.
1886—87. FRANCIS POOLE LANSDOWN.
1887—88. LEMUEL MATTHEWS GRIFFITHS.
1888—89. ROBERT SHINGLETON SMITH, M.D., B.Sc. Lond.
1889—90. NELSON CONGREVE DOBSON, Ch.M. Brist.
1890—91. SAMUEL HENRY SWAYNE.
1891—92. FRANCIS RICHARDSON CROSS, M.B. Lond., LL.D. Brist.
1892—93. EDWARD MARKHAM SKERRITT, M.D., B.S., B.A. Lond.
1893—94. JAMES GREIG SMITH, M.B., C.M., M.A. Aberd., F.R.S.E.
1894—95. ALFRED JAMES HARRISON, M.B. Lond.
1895—96. ARTHUR WILLIAM PRICHARD.
1896—97. ALFRED EDWARD AUST LAWRENCE, M.D., C.M. Aberd.
1897—98. JOHN EDWARD SHAW, M.B., C.M. Ed.
1898—99. ROBERT ROXBURGH, M.B. Ed.
1899—00. WILLIAM HENRY HARSANT.
1900—01. DAVID SAMUEL DAVIES, M.D. Lond.
1901—02. BARCLAY JOSIAH BARON, M.B., C.M. Ed.
1902—03. GEORGE MUNRO SMITH, M.D. Brist.
1903—04. JAMES PAUL BUSH, C.M.G., C.B.E., Ch.M. Brist
1904—05. REGINALD EAGER, M.D. Lond.
1905—06. JOHN DACRE.
1906—07. JAMES TAYLOR.
1907—08. HENRY WALDO, M.D., C.M. Aberd.
1908—09. JOHN MICHELL CLARKE, M.D., M.A. Cantab.
1909—10. JAMES SWAIN, C.B., C.B.E., M.D., M.S. Lond.
1910—11. BERTRAM MITFORD HERON ROGERS, M.D., B.Ch., B.A. Oxon.
1911—12. CHARLES ALEXANDER MORTON.
1912—13. WALTER CARLESS SWAYNE, M.D., B.S. Lond. and Brist.
1913—14. PATRICK WATSON-WILLIAMS, M.D. Lond., M.D. (Hon. Causa) Brist.
1914—15. WILLIAM HARRY CHRISTOPHER NEWNHAM, M.A., M.B. Cantab.
1915—16. GEORGE PARKER, M.A., M.D. Cantab., LL.D. Brist.
1916—17. FRANCIS HENRY EDGEWORTH, M.A., M.D. Cantab., D.Sc. Lond.
1917—18. FREDERICK LACE.
1918—19. ROBERT GUTHRIE POOLE LANSDOWN, M.D., B.S. Durh.
1919—20. I. WALKER HALL, M.D., Ch.B. Vict.
1920—21. LEOPOLD ERNEST VALENTINE EVERY-CLAYTON, M.D. Lond.
1921—22. CYRIL HUTCHINSON WALKER, B.A., M.B. Cantab.
1922—23. JOHN ALEXANDER NIXON, C.M.G., B.A., M.D. Cantab.
1923—24. JOHN LACY FIRTH, M.D., M.S. Lond.
1924—25. JOHN ODERY SYMES, M.D. Lond.
1925—26. THOMAS CARWARDINE, M.S. Lond.
1926—27. ARTHUR LAUNCELOT FLEMMING, M.B., Ch.B. Brist.
1927—28. WALTER KENNETH WILLS, M.A., M.B., B.Ch. Cantab.
1928—29. HENRY LAWRENCE ORMEROD, M.D., B.Ch. R.U.I.
1929—30. CHARLES FERRIER WALTERS.
1930—31. DAVID CHARLES RAYNER, Ch.M. Brist.
1931—32. JOHN ROGER CHARLES, M.A., M.D. Cantab.
1932—33. ERNEST WILLIAM HEY GROVES, M.D., M.S., D.Sc., B.Sc. Lond.
1933—34. HERBERT ELWIN HARRIS, B.A., M.B. Cantab.
1934—35. A. RENDLE SHORT, B.Sc., M.D. Lond.
1935—36. ARTHUR ERNEST ILES, O.B.E., M.B., B.S. Lond.
1936—37. RICHARD CHRISTOPHER CLARKE, O.B.E, M.B. Lond.
1937—38. CLIFFORD A. MOORE, M.B., M.S. Lond.
1938—40. LEONARD AUGUSTUS MOORE, M.B., Ch.B. Brist.
1940—41. CHARLES CORFIELD, M.D. Durh., Barr.-at-Law.

1941.

### LIST OF SUBSCRIBERS

TO THE

### Bristol Medico-Chirurgical Journal.

### HONORARY MEMBER OF THE SOCIETY.

Swain, James, *C.B.*, *C.B.E.*, M.D., M.S. Lond. .. .. .. .. Wyndham House, Easton-in-Gordano.

### SUBSCRIBERS.

*Those marked * are Members of the Society.*

| | |
|---|---|
| *Adams, A. W., M.S. Lond. .. .. .. .. | Rodney House, Clifton, Bristol 8. |
| Adams, J. B. .. .. .. .. .. .. .. .. | Kent Lodge, Eastbourne. |
| *Adams, S.B., Ph.D., M.B., Ch.B. Brist. .. .. | 19 Charlotte Street, Bristol 1. |
| *Aldwinckle, Helen M., M.B., Ch.B. Brist. .. | 23 Rockland Road, Downend, Bristol. |
| *Alexander, A. J. P., M.D. Belf. .. .. .. | Winsley Sanatorium, near Bath. |
| *Alexander, D. A., M.B., Ch.B. Vict. .. .. | 112 Pembroke Road, Clifton, Bristol 8. |
| Alexander, G. L., B.A., M.B., B.Ch. Cantab. | West African Medical Service, Laura, via Accra, Gold Coast. |
| *Alford, A. F., M.B., Ch.B. Brist. .. .. .. | Board of Education, Durley Dene Hotel, Bournemouth. |
| Alford, H. J., M.D. Lond. .. .. .. .. | 4 Park Terrace, Taunton. |
| Ashton, L. P., M.B., Ch.B. Brist. .. .. .. | Kapsowar, P.O. Eldoret, Kenya, East Africa. |
| *Aubrey, T., M.B. Lond. .. .. .. .. .. | Bitton, near Bristol. |
| *Bain, W. .. .. .. .. .. .. .. .. .. | 1 Greville Road, Southville, Bristol 3. |
| *Baker, Lily A., M.D., B.Ch., B.A.O., R.U.I. | 23 Pembroke Road, Clifton, Bristol 8. |
| *Ball, Jean, M.B., Ch.B. Brist. .. .. .. | Succoth, Duckmoor Road, Ashton Gate, Bristol 3. |
| *Barber, M. C., M.B., Ch.B. Brist. .. .. .. | Ingledene, High Street, Staple Hill, Bristol. |
| *Barbour, R. F., M.A. Cantab., M.B., Ch.B. | 16 Mortimer Road, Clifton, Bristol. |
| *Barker, G. H., M.D., B.Sc. Lond. .. .. .. | 124 Redland Road, Bristol 6. |
| *Bates, R. M. .. .. .. .. .. .. .. .. | Purdown House, Stapleton, Bristol 5. |
| *Baxter, Ursula, M.B., Ch.B. Brist. .. .. | 167 Wells Road, Knowle, Bristol 4. |
| *Baxter, V. T., M.B., Ch.B. .. .. .. .. | 167 Wells Road, Knowle, Bristol 4. |
| *Beatson, D. H., M.B., Ch.B. Brist. .. .. | 8 Richmond Park Road, Clifton, Bristol 8. |
| *Bell, R. J. Irving .. .. .. .. .. .. .. | 5a Oakfield Road, Clifton, Bristol 8. |
| *Bergin, F. G. .. .. .. .. .. .. .. .. | 31 Oakfield Road, Clifton, Bristol 8. |
| *Berry, R. J. A., Prof., M.D., C.M. Edin. .. | 320 Canford Lane, Bristol 9. |
| *Birrell, J. A., M.D., B.S. Lond. .. .. .. | 1 Victoria Square, Bristol 8. |
| Blackett, J. F., M.D., B.S. Lond. .. .. .. | Hamsterley, Bloomfield Park, Bath. |
| Bletchly, G. Playne, M.B. Lond. .. .. | c/o Lloyds Bank, Nailsworth, Glos. |
| *Bodman, A. P., M.B., Ch.B. .. .. .. .. | 11 Hurle Crescent, Clifton, Bristol 8. |
| *Bodman, F. H., M.D., Ch.B. Brist. .. .. .. | 2 Redland Court Road, Bristol 6. |
| Bolt, R. F. .. .. .. .. .. .. .. .. .. | 70 Great Russell Street, London, W.C.1. |
| *Boulton, B. J., M.B., Ch.B. Brist. .. .. | Ham Green Hospital, Pill, near Bristol. |
| *Bown, Naomi J., M.B., Ch.B. Brist. .. .. | The Corner, Nailsea, Somerset. |
| *Bradbeer, E. G., M.B., Ch.B. Brist. .. .. | 13 Percival Road, Bristol 8. |
| Brasher, C. W. J. .. .. .. .. .. .. .. | 62 Queen Anne St., Cavendish Square, London, W.1. |
| *Brinckman, Winifred P., M.B., Ch.B. Brist. | 4 Oakland Road, Redland, Bristol 6. |
| *Britton, R. B., M.B., B.S. Lond. .. .. .. | 33a Whiteladies Road, Clifton, Bristol 8. |
| *Brocklehurst, R. J., M.A., D.M. Oxon. .. | The University, Bristol 8. |
| Brown, J. E., M.B., Ch.B. Brist. .. .. .. | Coulthurst Villa, Brives Road, Maraval, Trinidad, B.W.I. |
| *Burges, R. .. .. .. .. .. .. .. .. .. | Druid's Mead, Stoke Bishop, Bristol 9. |
| *Bush, G. B., M.B., Ch.B. Brist. .. .. .. | Litfield House, Clifton, Bristol 8. |

| | |
|---|---|
| *CAIRNS, F. J. | Devon House, Whitehall Road, Bristol 5. |
| *CAPPER, W. M., M.B., B.S. Lond. | Bristol Royal Infirmary, Bristol. |
| *CAREY, R. S., *O.B.E.*, B.A. Cantab. | 502 Bath Road, Brislington, Bristol 4. |
| *CARLETON, H. H., M.A., M.D., B.Ch. Oxon. | 1 Lansdown Road, Clifton, Bristol 8. |
| *CASSON, E., M.D., Ch.B. Brist. | Dorset House, Clifton Down, Bristol 8. |
| *CATES, H. J., M.D. Lond. | Northwoods House, Winterbourne, Bristol. |
| *CHAMBERS, E. R. | 9 Clifton Park, Clifton, Bristol 8. |
| *CHARLES, J. R., M.A., M.D. Cantab. | Litfield House, Clifton Down, Bristol 8. |
| *CHITTY, H., M.S. Lond. | Naish House, Clapton-in-Gordano. |
| *CHRISTOFFERSON, P. E., M.B., Ch.B. Brist. | 4 Victoria Square, Clifton, Bristol 8. |
| *CLAREMONT, L. E., M.D.S. Brist. | 5 Rodney Place, Clifton, Bristol 8. |
| *CLARK, DENNIS O. | 168 Milton Road, Bristol Road, Weston-super-Mare. |
| *CLARKE, R. C., *O.B.E.*, M.B., Ch.B. Brist. | 29 Victoria Square, Clifton, Bristol 8. |
| *CLUTTERBUCK, E. R., M.B., Ch.B. Brist. | 63 Walliscote Road, Weston-super-Mare. |
| *COATES, H. | Notts County Mental Hospital, Radcliffe-on-Trent. |
| *COCHRANE, J. H. | 11 Bryant's Hill, St. George, Bristol 5. |
| *COLLINGWOOD, F. C., M.B., Ch.B. Brist. | 7 Hurle Crescent, Clifton, Bristol 8. |
| *COOKE, ELIZABETH M., M.D. Lond. | Failand Lodge, Guthrie Road, Clifton. |
| *COOKE, R. V., Ch.M. Brist. | Failand Lodge, Guthrie Road, Clifton. |
| *COOKSON, R. G. | 5 Worcester Road, Clifton, Bristol 8. |
| COOMBS, N. C. | Romaldkirk, Barnard Castle, Co. Durham. |
| *COOPER, W. R. | 13 Westfield Park, Redland, Bristol 6. |
| *CORFIELD, C., M.D. Durh. | 5 Kensington Place, Clifton, Bristol 8. |
| *CORNER, BERYL, M.D., B.S. Lond. | 3 Elton House, Rodney Place, Bristol 8. |
| *COSSHAM, W. L., M.B., Ch.B. Brist. | 3 Claremont Road, Bishopston, Bristol 7. |
| *COURTNEY, R. M., M.B., Ch.B. Glasg. | Manor House, Southmead Road, Bristol |
| *COXON, BEATRICE | 16 Richmond Hill, Clifton, Bristol 8. |
| *CRAIG, ANNE A., M.D., B.S. Lond. | 35 Queen's Court, Clifton, Bristol 8. |
| *CROOK, B. A., M.B., Ch.B. Brist. | The Laurels, Timsbury, near Bath. |
| *CROSSMAN, F. W., M.B. Durh. | White's Hill, Hambrook, near Bristol. |
| *DATTA, S. S., M.D. Brist. | Snowdon House, Fishponds, Bristol. |
| *DAVIES, E. D. D. | Standish House, Stonehouse, Glos. |
| *DAVIES, J. N. P., M.B., Ch.B. | Ham Green Hospital, near Bristol. |
| *DAWSON, W. R., *O.B.E.*, M.D. Dub. | 18 Brock Street, Bath. |
| *DELANEY, N., M.B., Ch.B. Manch. | The Meade, Bishopsworth, Somerset. |
| *DICKSON, W. A., M.D. St. And. | Standish House, Stonehouse, Glos. |
| *DISNEY, M., M.D. Lond. | Bristol Royal Infirmary. |
| *DIX, C. | 14 Mortimer Road, Clifton, Bristol 8. |
| *DIXON, T. B. | 11 Pembroke Road, Clifton, Bristol 8. |
| *DODD, GRANTHAM, M.B., B.S. | 42 Woodstock Road, Bristol 6. |
| *DUERDEN, J. R., M.B., Ch.B. Brist. | 4 Cecil Road, Bristol 8. |
| *DUTTON, HILDA R. | 405 Stapleton Road, Bristol 5. |
| *DYER, W. P. | West View, Westbury-on-Trym, Bristol. |
| *EGLINTON, J. H. C. | Street, Somerset. |
| ELLIOTT, F. P., M.B., C.M. Aberd. | 113 Grove Road, Walthamstow, London, E.17. |
| *ELLIOTT, W. H. A., M.B., B.S. Lond. | 116 Pembroke Road, Clifton, Bristol 8. |
| *ELLIS, W. T. | Tregenna House, Shirehampton. |
| *EMERY, J. L. | Childrens' Hospital 2. |
| *EVANS, G. M., M.B., Ch.B. Brist. | 117 Ashley Road, Bristol. |
| *EYRE-BROOK, A. L., M.B., M.S. Lond. | The Cottage, Portishead. |
| *FALLON, Col. J. | 35 Henleaze Gardens, Henleaze, Bristol. |
| *FAULL, J. L. | 61 Cotham Brow, Bristol 6. |
| *FAWN, G. F. | 6 Oakfield Road, Clifton, Bristol 8. |
| *FELLS, G. R., M.B., Ch.B. Brist. | 19 Southmead Road, Bristol. |
| *FELLS, R. R., M.B., B.S. Lond. | 17 Mortimer Road, Clifton, Bristol 8. |
| *FENELEY, G. L., M.B., Ch.B. Brist. | Englewood, Falcondale Road, Westbury-on-Trym. |
| *FINZEL, H. F. M., M.D. Brist. | Nare House, Birchwood Road, St. Anne's Park, Bristol 4. |
| *FIRTH, J. L., M.D., M.S. Lond. | 8 Victoria Square, Clifton, Bristol 8. |
| *FISHER, A. C., M.D. Brist. | Roan Antelope Mine, N. Rhodesia. |

*FITZGIBBON, G. M., M.D. Lond. .. .. .. 75 Pembroke Road, Clifton, Bristol 8.
*FITZGIBBON, L. PRISCILLA, M.B., B.S. Lond. .. 75 Pembroke Road, Clifton, Bristol 8.
*FORRESTER-BROWN, MAUD, M.D., M.S. Lond. 22 Combe Park, Bath.
*FOSS, G. L., M.B., B.Ch. Cantab. .. .. .. Cloud's Hill House, St. George, Bristol 5.
*FOX, F. E., B.A. Cantab. .. .. .. .. .. Brislington House, Brislington, Bristol 4.
*FRASER, A. D., M.A., M.B., Ch.B. Glasg. .. 4 Alexandra Road, Clifton, Bristol 8.
*FRASER, D., M.B., Ch.B. Edin. .. .. .. Westgate Dursley, Glos.
*FULLER, H. L. .. .. .. .. .. .. .. .. 19 The Circus, Bath.
*FURNIVAL, Col. C. H. .. .. .. .. .. .. "Yatton," Wellington Hill Horfield, Bristol 7.

*GARDEN, R. R., M.A., M.B., B.Ch. Aberd. .. 9 Harley Place, Clifton, Bristol 8.
GARLAND, E. B., M.B., C.M. Edin. .. .. .. 114a Hampton Road, Redland, Bristol 6.
*GERRISH, NORMAN, M.B., Ch.B. Brist. .. .. 2 Station Road, Keynsham, Som.
*GIBB, HELEN M., M.B., Ch.B. Glasgow .. .. 10 Hill View, Henleaze, Bristol.
*GIBBS, J. R. .. .. .. .. .. .. .. .. 48 Codrington Road, Bishopston, Bristol 7.
*GORHAM, A. P., M.B., Ch.B. Brist. .. .. .. 53 Westbury Road, Westbury-on-Trym.
*GORNALL, W. A., M.D. Brist. .. .. .. .. Ulvik, Nailsea, near Bristol.
*GREEN, S. B., M.B. Lond. .. .. .. .. .. The Black Wicket, Westbury-on-Trym.
*GREENLAW, MADGE E., M.B., Ch.B. Brist. .. 206 Redland Road, Bristol 6.
GRIFFITHS, CORNELIUS A. .. .. .. .. .. 35 Newport Road, Cardiff.
*GROVES, E. W. HEY, M.D., M.S., D.Sc. Lond. .. 25 Victoria Square, Clifton, Bristol 8.

*HALL, E. GEORGE, M.B. Lond. .. .. .. .. 42 Apsley Road, Clifton, Bristol 8.
*HAM, C. I. M.B., Ch.B. Brist. .. .. .. .. Frenchay Park Sanatorium, Frenchay, near Bristol.
*HARRIS, H. E., M.A., M.B., B.Ch. Cantab. .. 13 Lansdown Place, Clifton, Bristol 8.
*HARTLEY, GRACE, M.B., B.S. Lond. .. .. 49 Queen's Court, Clifton, Bristol 8.
*HEALES, J. N., M.B., Ch.B. Brist. .. .. .. Holmcroft, Street, Somerset.
*HECTOR, F., M.D. Lond. .. .. .. .. .. 1 Victoria Square, Clifton, Bristol 8.
*HEMPHILL, R., M.D. Dub. .. .. .. .. 90 Cleeve Hill, Downend, near Bristol.
*HENDERSON, JAMES, M.B., Ch.B. Aberd. .. The City Sanatorium, Yardley Road, Birmingham.
*HERAPATH, C. E. K., *M.C.*, M.D. Lond. .. Ormlie, Clifton, Bristol 8.
*HERON, GORDON, M.B., Ch.B. Brist. .. .. 26 Henbury Road, Westbury-on-Trym, Bristol.
*HERON, DORIS, M.B., Ch.B. Brist. .. .. .. 26 Henbury Road, Westbury-on-Trym, Bristol.
*HEWER, Professor T. F., M.D. Brist. .. .. Canynge Hall, Bristol 8.
*HILL, WINIFRED, M.B., Ch.B. Brist. .. .. c/o Lloyd's Bank, Ilford.
*HOFFMAN, H. LOVELL, B.A., M.B., B.Ch. Cantab. 26 The Circus, Bath.
*HOLLAND, W. A. L. .. .. .. .. .. .. 10 Brunswick Square, Bristol.
*HOOKER, J. A., M.B., Ch.B. Brist. .. .. .. 16 St. Edyth's Road, Sea Mills 9.
*HOSKEN, J. G. F... .. .. .. .. .. .. Uley, Glos.
HOSPITAL, BRISTOL GENERAL (*Secretary*, T. W. GREGG).
*HOUGHTON, LYDIA M., M.B., B.S. Lond. .. 74 Church Road, Redfield, Bristol 5.
*HOWARD, C. R. GRENVILLE, M.B. .. .. .. Royal Navy.
*HOWELL, H. .. .. .. .. .. .. .. .. Kincraig, Beach Road, Weston-super-Mare.
*HOWELL, T. E. .. .. .. .. .. .. .. 6 Richmond Hill, Bristol 8.
*HUGHES, F. W. TERRELL, M.B., Ch.B. Brist. Chew Magna, Somerset.
*HUGHES, MARGUERITE G., M.B., Ch.B. Brist. 12 Upper Belgrave Road, Clifton, Bristol 8.
*HUNTER, F. S. .. .. .. .. .. .. .. c/o Messrs. J. Wright & Sons Ltd., Publishers, Bristol 1.
*HYATT, ANNIE W., M.B., B.S. Lond. .. .. Merrymead, Shepton Mallet, Som.

*ILES, ANNA, M.B., Ch.B. Brist. .. .. .. 87 Pembroke Road, Clifton, Bristol 8.
*ILES, A. E., *O.B.E.*, M.B., B.S. Lond. .. .. 87 Pembroke Road, Clifton, Bristol 8.
INFIRMARY, BRISTOL ROYAL (*Secretary*, E. C. SMITH).
INGLE, C. DURBAN .. .. .. .. .. .. .. Somerton, Somerset.
*ISSERLIN, B. .. .. .. .. .. .. .. .. 2 College Road, Bristol 8.

*JACKMAN, W. A., M.B., Ch.B. Brist. .. .. 15 Mortimer Road, Clifton, Bristol 8.
*JAMES, APRIL D., M.B., Ch.B. Brist. .. .. (Address unknown.)
*JAMES, J. A., M.D. Lond... .. .. .. .. Litfield House, Clifton Down, Bristol 8.
*JAMES, J. A., Senr. .. .. .. .. .. .. 43 Nevil Road, Bishopston, Bristol.

| | |
|---|---|
| *JENKINS, F. G., M.B., Ch.B. Brist. .. .. .. | 51 Redcliff Hill, Bristol 1. |
| *JENKINS, R. D. .. .. .. .. .. .. .. .. .. | The Copper Beeches, Almondsbury, near Bristol. |
| *JENKINS, P. K., M.B., Ch.B. .. .. .. .. .. | R.A.M.C. |
| *JOHNSON, R. G., M.B. Lond. .. .. .. .. .. | 119 Redland Road, Bristol 6. |
| *JOLL, C. A., M.D., M.S. Lond. .. .. .. .. | 64 Harley Street, London, W.1. |
| *JONES, MEGAN E. .. .. .. .. .. .. .. .. | Royal Infirmary, Bristol 2. |
| *JORDAN, L. R., M.B., Ch.B. Brist. .. .. .. | National Temperance Hospital, Hampstead Road, N.W.1. |
| *JOSCELYNE, P. C., M.B., Ch.B. Brist. .. .. | 17 Falcondale Road, Westbury-on-Trym. |
| *KEMM, N. F., M.B., B.S. Lond. .. .. .. | 200 Redland Road, Durdham Park, Bristol, 6. |
| *KERSLEY, G. D., M.D. .. .. .. .. .. .. | 6 The Circus, Bath. |
| KING, E. F. .. .. .. .. .. .. .. .. .. .. .. | 2 Upper Wimpole Street, London, W.1. |
| *KINGSTON, S. H., M.B., Ch.B. Brist. .. .. | 3 Whiteladies Road, Clifton, Bristol 8. |
| *KYLE, H. G., M.A., M.D., B.Ch. Oxon. .. .. | 31 Westbury Road, Bristol. |
| *LACE, F .. .. .. .. .. .. .. .. .. .. .. | 5 Gay Street, Bath. |
| LALONDE, T. P., M.B., Ch.B. Brist... .. .. | Wykeham House, Romsey, Hants. |
| *LEES, E. LEONARD, M.D., C.M. Edin. .. .. | 28 Downs Park West, Bristol 6. |
| *LEGAT, R. EDDOWES, M.B., C.M. Edin. .. .. | 1 Cambridge Mansions. Woodland Road, Bristol 6. |
| *LEIGHTON, S. T. P., O.B.E., M.B., Ch.B. Vict. | Lamport Court, Stinchcombe, Glos. |
| *LEWIS, F. J., M.B., Ch.B. Brist. .. .. .. | Canynge Hall, Bristol 8. |
| *LING, T. M., M.A., B.M., B.Ch. .. .. .. | 50 Pembroke Road, Bristol 8. |
| *LINTON, MARION S., M.B. Lond. .. .. .. | 1 Brecon Road, Henleaze, Bristol. |
| *LISTER, A. E. J., M.B., B.S. Lond. .. .. | 86 Pembroke Road, Clifton, Bristol 8. |
| *LISTER, L. MARGARET .. .. .. .. .. .. | 12 All Saints Road, Clifton, Bristol 8. |
| *LOGAN, H. B. .. .. .. .. .. .. .. .. .. | Elmwood, Aberdeen Road, Cotham, Bristol 6. |
| *LOXTON, S. D., M.B., Ch.B. .. .. .. .. .. | 17 Oakfield Grove, Clifton, Bristol 8. |
| *LUCAS, J. E... .. .. .. .. .. .. .. .. .. | c/o 132 York Road, Bristol 3. |
| *LUCAS, J. J. S., M.D. Lond. .. .. .. .. | 186 Wells Road, Knowle, Bristol 4. |
| *LUCAS, J. V... .. .. .. .. .. .. .. .. .. | 186 Wells Road, Knowle, Bristol 4. |
| *LUCAS R. P., M.B., Ch.B. Brist. .. .. .. | 15 Edgecumbe Road, Redland Bristol 6. |
| *MCCREA, PHILLIP, M.D., B.Ch. Dub. .. .. | 56 Kellaway Avenue, Bristol 6. |
| *MCKENZIE, W. G., *M.C.* .. .. .. .. .. .. | 10 Woodland Road, Surbiton. |
| *MACLACHLAN, A. M., M.B., Ch.B. Glasg. .. | 65 Redcliff Hill, Bristol 1. |
| *MARWOOD, S. F., M.D., B.S. .. .. .. .. .. | Troy House, Kingswood, Bristol. |
| *MCLANNAHAN, J. G. .. .. .. .. .. .. .. .. | The Mount, Stonehouse. |
| *MACLAREN, CATHERINE J., M.B., Ch.B. Edin. | Hereford House, Clifton Down. |
| MACLEAN, SIR EWEN J., M.D., C.M. Edin. .. | 12 Park Place, Cardiff. |
| *MACLEOD, G., M.A. Glas., M.D., M.B. .. .. | Wargrave, Clevedon, Somerset. |
| *MARSHALL, A. H., M.B., Ch.B. Brist. .. .. | Odelos, Canford Lane, Bristol. |
| MARSHALL, A. L., M.B., B.A. Cantab. .. .. | Laxfield, Woodbridge, Suffolk. |
| *MARTIN, J. E., M.B., Ch.B. .. .. .. .. .. | 32 Whiteladies Road, Clifton. |
| *MARTIN, J. J. B., M.A., M.D. Edin... .. .. | Bristol Mental Hospital. |
| *MARTIN, P. S., *M.C.* .. .. .. .. .. .. .. | Victoria Quadrant, Weston-super-Mare. |
| *MATHESON, N. R., M.B., Ch.B... .. .. .. .. | 37 Abbey Road, Westbury-on-Trym. |
| MIALL, C. L'O .. .. .. .. .. .. .. .. .. | Elm Hayes, Paulton, near Bristol. |
| *MILLING, T., M.B., Ch.B. Belf. .. .. .. .. | 1 Vicarage Road, Southville, Bristol 3. |
| *MOORE, C. A., M.B., M.S. Lond. .. .. .. | South Lodge, Kingsweston Road, Henbury Bristol. |
| *MOORE, L. A., M.B., Ch.B. Brist. .. .. .. | Hillsborough, Cotham Park, Bristol 6. |
| *MOORE, R. H., M.B., Ch.B. Brist. .. .. .. | White Cross Court, Wells Road, Bristol 4. |
| *MORGANS, C. C., M.B., Ch.B. Cantab. .. .. | 65 Lower Redland Road, Bristol 6. |
| *MORRIS, L. N., M.B., Ch.B. Brist. .. .. .. | 13 Belgrave Road, Clifton, Bristol 8. |
| *MUNDY, G. S., M.B., B.Ch. Brist. .. .. .. | Homewood, Coombe Dingle, Bristol. |
| *MYLES, Q. St. L., M.A., B.M., B.Ch. Oxon. .. | 7 Apsley Road, Clifton, Bristol 8. |
| *NEWLANDS, H. J. M.B., Ch.B... .. .. .. | Moray House, Fishponds, Bristol. |
| *NICHOLSON-LAILEY, J. R. .. .. .. .. .. .. | Elmfield House, South Road, Taunton. |
| *NIXON, DOREEN, M.B., B.S. Lond. .. .. .. | 7 Lansdown Place, Clifton, Bristol 8. |
| *NIXON, J. A., *C.M.G.*, M.D. Cantab. .. .. | 7 Lansdown Place, Clifton, Bristol 8. |

*NOBLE, J. I., M.B., Ch.B. Liverp. .. .. .. 102 Pembroke Road, Clifton, Bristol 8.
*NORMAN, R. M., M.D. Brist. .. .. .. .. Highwood, Stapleton Park, Bristol.
*NOTT, WINIFRED, M.B., Ch.B. Brist. .. .. Fassiefern, 8 Rylestone Grove, Stoke Bishop, Bristol 9.

*O'DONOHOE, MONICA A., M.B., Ch.B. .. .. 1 Beaconsfield Road, Bristol 8.
*ORMEROD, G. L. M.A., M.B., B.Ch. Cantab .. 2 Henleaze Road, Westbury-on-Trym, Bristol.
*ORR-EWING, H. J., *M.C.*, M.D. Lond. .. .. 1 Beaufort Buildings, Clifton, Bristol 8.

*PALIN, A. G., B.M., B.Ch. Oxon. .. .. .. Litfield House, Clifton Down, Bristol 8.
*PARKES, J. A., M.B., Ch.B. Liverp. .. .. 8 South Road, Redland, Bristol 6.
*PARKES, P. W. J. .. .. .. .. .. .. .. 164 Filton Avenue, Bristol 7.
PARKINSON, Lt.-Col. G. S., *D.S.O.*, R.A.M.C. London School of Hygiene and Tropical Medicine, Keppel Street, London, W.C. 1.
*PARRY, R. H., M.B., B.S. Lond. .. .. .. Public Health Offices, 40 Prince Street, Bristol 1.
PARSONS, Sir J. H., M.B., B.S., B.Sc. Lond... 54 Queen Anne Street, London, W.
*PAUL, R. G. .. .. .. .. .. .. .. .. .. 23 Pembroke Road, Bristol 8.
*PAULI, C. H... .. .. .. .. .. .. .. .. Pagan's Hill Farm, Chew Stoke.
*PERRY, B., M.D., Ch.B. Brist. .. .. .. .. 4 Richmond Park Road, Clifton, Bristol 8.
*PHILLIPS, P., M.B., Ch.B. Brist. .. .. .. Southmead Hospital, Bristol.
*PIRRIE, A. L., M.B., Ch.B. Glasg. .. .. 528 Kensington Hill, Bristol.
*POCOCK, J. A., M.A., M.B., B.Ch. Cantab. .. General Hospital, Bristol.
PORTER, C. P. .. .. .. .. .. .. .. .. 28 Mill Street, Kidderminster.
*POTTER, MABEL F., M.B., Ch.B. Brist. .. .. 79 Pembroke Road, Clifton, Bristol 8.
*POWELL, H. F., M.D. Brux. .. .. .. .. Ellenborough Park, Weston-super-Mare.
*PRICE, C. H. G., M.B., Ch.B. Brist. .. .. 6 Lansdown Place, Bristol 8.
*PRICE, N. L., M.B., Ch.B. Brist. .. .. .. Litfield House, Clifton Down, Bristol 8.
*PRIDIE, K. H., M.B., B.S. Lond. .. .. .. 58 St. John's Road, Clifton, Bristol 8.
*PROWSE, D. C., M.B., Ch.B. Brist. .. .. .. Wigmore House, Thornbury, near Bristol.
*PYKE, H. D., M.B., Ch.B. Brist. .. .. .. 88 Redland Road, Bristol 6.

*RANKIN, P. C., M.B., Ch.B. Glasg. .. .. .. Lawrence Hill House, Bristol.
*RAYNER, D. C., Ch.M. Brist. .. .. .. .. 9 Lansdown Place, Clifton, Bristol 8.
*REDMAN, ETHEL, M.B., Ch.B. .. .. .. .. 55 Bridgwater Road, Bedminster Down, Bristol 3.
*ROBERTS, J. A. F., M.A. Cantab., M.B., Ch.B., D.Sc. Edin. Stoke Park Colony, Bristol.
*ROBERTSON, D., M.B., Ch.B. Edin. .. .. .. 2 Knowle Road, Knowle, Bristol 4.
*ROBERTSON, L. G., M.B., Ch.B. .. .. .. 162 Redland Road, Bristol 6.
*ROGERS, H., M.D. Brist. .. .. .. .. .. 91 Old Park Ridings, Grange Park, N.21.
*ROLFE, R., B.A., M.B., B.C. Cantab. .. .. Park House, St. Andrew's Road, Avonmouth.
*ROWLANDS, R. D. .. .. .. .. .. .. .. Elmfield House, South Road, Taunton, Som.
*ROWLEY, A. T. .. .. .. .. .. .. .. .. 13 Walliscote Road, Weston-super-Mare.
*RUDOLF, MAJOR G. DE M. .. .. .. .. R.A.M.C.
*RYAN, P. B., M.B., Ch.B... .. .. .. .. Southmead Hospital, Bristol.

*SAMPSON, O. E. L., M.B., Ch.B. Brist. .. .. 77 Stackpool Road, Bristol 3.
*SCARFF, G., M.B., Ch.B. Edin. .. .. .. .. 83 Pembroke Road, Clifton, Bristol 8.
*SHEPHERD, H. L., M.B., Ch.M. Brist. .. .. 79 Pembroke Road, Clifton, Bristol 8.
*SHORT, A. R., B.Sc., M.D., B.S. Lond. .. .. 69 Pembroke Road, Clifton, Bristol 8.
*SMITH, T. W., M.D. Lond... .. .. .. .. 26 The Circus, Bath.
*SMYTHE, H. J. D., *M.C.*, M.D., M.S. Lond. .. 15 Eaton Crescent, Clifton, Bristol 8.
*SNAWDON, J. W. E., M.B., Ch.B., B.D.S. .. 170 Redland Road, Bristol 6.
*SOLTAU, H. K. V., M.D. Lond. .. .. .. 19 The Avenue, Clifton, Bristol 8.
*SOMERS, MARY A. E. .. .. .. .. .. .. 2 Ashcombe Road, Weston-super-Mare.
*SPOOR, A., M.B., B.Ch. Cantab. .. .. .. The Hydro, Bristol 1.
*STEVEN, G. D., M.B., Ch.B. Edin., D.P.H. .. 6 Upper Church Street, Bath.
*STEPHEN, R. J. .. .. .. .. .. .. .. .. 92 Redland Road, Bristol 6.
STONE, DORIS M., M.D., B.S. Lond. .. .. Beechwood, Church Street, Epsom.
*STROVER, H. W. M., *O.B.E.*, M.B., Ch.B. Aberd. 90 Redland Road, Bristol 6.
*STRUTHERS, A. J., M.B., Ch.B. Glasg. .. .. 100 Church Road, Redfield, Bristol 5.
*STRUTHERS, GEO., M.B., Ch.B. Glasgow.. .. 100 Church Road, Bristol 5.
*SUTTON, G. E. F., M.D. Lond. .. .. .. .. 1 Pembroke Road, Clifton, Bristol 8.
*SYMES, J. O., M.D. Lond. .. .. .. .. .. 6 Pembroke Vale, Clifton, Bristol 8.

| | |
|---|---|
| *TALLACK, R. J. K., M.B., Ch.B. .. .. .. .. | 9 Christchurch Road, Clifton, Bristol 8. |
| *TASKER, D. G. C., M.B., M.S. Lond. .. .. | 9 College Fields, Clifton, Bristol 8. |
| *TAYLOR, A. L., M.D. Lond. .. .. .. .. | The General Hospital, Bristol 1. |
| TAYLOR, A. L. .. .. .. .. .. .. .. .. .. | The Royal Infirmary, Bristol 2. |
| THIN, J. .. .. .. .. .. .. .. .. .. .. .. | 54 and 55 South Bridge, Edinburgh. |
| *TODD, A. T., *O.B.E.*, M.D. Edin. .. .. .. | Royal Park House, Clifton, Bristol 8. |
| TRINICK, RICHARD H. .. .. .. .. .. .. .. | 42 Cherry Valley Gardens, Belfast. |
| TRIPP, DOROTHY M. H. .. .. .. .. .. .. | Mission Hospital, Karim Nagar, Nizam's Dominions, South India. |
| *TRIST, J. R. R., *M.C.* .. .. .. .. .. .. | 120 Henleaze Road, Henleaze. |
| *TRYON, VICTORIA S., M.B., Ch.B. Brist. .. | 3 Harley Place, Clifton, Bristol 8. |
| | |
| *WAGSTAFFE, E. M., M.B., Ch.B. .. .. .. .. | Winford Orthopædic Hospital. |
| *WANSBROUGH, T. B., M.B., Ch.B. Brist. .. | 23 Pembroke Road, Clifton, Bristol 8. |
| *WALKER, CYRIL H., B.A., M.B. Cantab. .. | Harewood, Clapton-in-Gordano, Som. |
| *WALTERS, C. F. .. .. .. .. .. .. .. .. .. | Downover, Walton Down, near Clevedon. |
| WAKE, S. C., M.B., Ch.B. Brist. .. .. .. | "Stanhope," Bideford, N. Devon. |
| *WARD, H. W. .. .. .. .. .. .. .. .. .. | Chipping Manor, Wotton-under-Edge. |
| *WARREN, R., M.A., M.D. Oxon. .. .. .. | Heathgates, Weston-super-Mare. |
| *WATSON-WILLIAMS, E., *M.C.*, B.A., M.B., Ch.M. Cantab. | 65 Pembroke Road, Clifton, Bristol 8. |
| WATTS, J. B. V. .. .. .. .. .. .. .. .. .. | Frantzina's Rust, Barberton, Transvaal, S. Africa. |
| WEDDELL, C. W. .. .. .. .. .. .. .. .. .. | Boydville, 46 Miller Street, West Melbourne, Australia. |
| *WIGODER, SYLVIA, M.A., M.D., Ph.D. .. .. | General Hospital, Bristol 1. |
| *WILBOND, R. G. .. .. .. .. .. .. .. .. .. | 76 Chesterfield Road, St. Andrew's Pk., Bristol. |
| *WILLIAMS, I., M.B., Ch.B. Brist. .. .. .. | "Ardrem," Hill View, Henleaze, Bristol. |
| *WILLIAMS, S. R., M.B. Lond. .. .. .. .. | Buckfastleigh, Devon. |
| *WILLWAY, F. W., M.D., M.S. Lond. .. .. | Bristol Royal Infirmary. |
| *WOOD, D. .. .. .. .. .. .. .. .. .. .. | 105 Pembroke Road, Clifton, Bristol 8. |
| *WOOD, URSULA, M.B., Ch.B. .. .. .. .. | Henley Lodge, Yatton, Som. |
| *WOOD, W. V., *M.C.*, B.A. Cantab. .. .. .. | Henley Lodge, Yatton, Som. |
| *WOODMAN, DOROTHY, M.D. Lond. .. .. .. | "Sherwood," Great Brockeridge, Westbury. |
| *WOOLLEY, W., M.B., Ch.B. Brist. .. .. .. | 239 Cranbrook Road, Redland. |
| *WORTHINGTON, LIEUT.-COL. F., M.B., Ch.B., Cantab. | Greytrees, Briercliffe Road, Coombe Dingle, Bristol 9. |
| *WRIGHT, A. J., M.B., B.S. Lond. .. .. .. | 2 Clifton Park, Clifton, Bristol 8. |
| *WRIGHT, WINIFRED M., M.B., B.S. Lond. .. | Merrymead, Shepton Mallet, Som. |
| | |
| *ZEALAND, L., M.D. Man. .. .. .. .. .. | Brecon Lodge, Westbury-on-Trym, Bristol. |

### *HONORARY VISITING MEMBERS.*

BRIDGES ,MAJOR ARTHUR BRODIE HAMILTON, R.A.M.C., *O.B.E.*

WARREN, SURGEON REAR-ADMIRAL LEONARD, *O.B.E.*, M.B., B.S. Lond.

WHITBY, COLONEL LIONEL ERNEST HOWARD, *A.M.S.*, *C.V.O.*, *M.C.*, M.D. Cantab.

WORTHINGTON, COLONEL FRANK, *A.M.S.*, *D.S.O.*,*O.B.E.*, M.B., B.Ch. Cantab.

And their staffs.

